# Development of a Compact Wireless Laplacian Electrode Module for Electromyograms and Its Human Interface Applications

**DOI:** 10.3390/s130202368

**Published:** 2013-02-08

**Authors:** Yutaka Fukuoka, Kenji Miyazawa, Hiroki Mori, Manabi Miyagi, Masafumi Nishida, Yasuo Horiuchi, Akira Ichikawa, Hiroshi Hoshino, Makoto Noshiro, Akinori Ueno

**Affiliations:** 1 Department of Electrical Engineering, Kogakuin University, Tokyo 163-8677, Japan; E-Mail: fukuoka@cc.kogakuin.ac.jp; 2 Master's Program in Electronic and Computer Engineering, Tokyo Denki University, Saitama 350-0394, Japan; E-Mail: ke-miya@ezweb.ne.jp; 3 Department of Electrical and Electronic Engineering, Utsunomiya University, Tochigi 321-8585, Japan; E-Mail: hiroki@speech-lab.org; 4 Research and Support Center on Higher Education for the Hearing Impaired and Visually Impaired, Tsukuba University of Technology, Ibaraki 305-8521, Japan; E-Mail: mmiyagi@k.tsukuba-tech.ac.jp; 5 Department of Information Systems Design, Doshisha University, Kyoto 610-0321, Japan; E-Mail: mnishida@mail.doshisha.ac.jp; 6 Graduate School of Science and Technology, Chiba University, Chiba 263-8522, Japan; E-Mail: hory@faculty.chiba-u.jp (Y.H.); ichikawa@1964.jukuin.keio.ac.jp (A.I.); 7 School of Science and Engineering, Tokyo Denki University, Saitama 350-0394, Japan; E-Mail: hoshinoh@pp.iij4u.or.jp; 8 Department of Clinical Engineering, School of Allied Health Sciences, Kitasato University, Kanagawa 228-8555, Japan; E-Mail: noshiromakoto@gmail.com; 9 Department of Electrical and Electronic Engineering, Tokyo Denki University, Tokyo 120-8551, Japan

**Keywords:** electromyogram (EMG), Laplacian derivation, wireless electrode module, Human-computer interface, technical aids for persons with physical disabilities, finger Braille, prosodic information

## Abstract

In this study, we developed a compact wireless Laplacian electrode module for electromyograms (EMGs). One of the advantages of the Laplacian electrode configuration is that EMGs obtained with it are expected to be sensitive to the firing of the muscle directly beneath the measurement site. The performance of the developed electrode module was investigated in two human interface applications: character-input interface and detection of finger movement during finger Braille typing. In the former application, the electrode module was combined with an EMG-mouse click converter circuit. In the latter, four electrode modules were used for detection of finger movements during finger Braille typing. Investigation on the character-input interface indicated that characters could be input stably by contraction of (a) the masseter, (b) trapezius, (c) *anterior tibialis* and (d) *flexor carpi ulnaris* muscles. This wide applicability is desirable when the interface is applied to persons with physical disabilities because the disability differs one to another. The investigation also demonstrated that the electrode module can work properly without any skin preparation. Finger movement detection experiments showed that each finger movement was more clearly detectable when comparing to EMGs recorded with conventional electrodes, suggesting that the Laplacian electrode module is more suitable for detecting the timing of finger movement during typing. This could be because the Laplacian configuration enables us to record EMGs just beneath the electrode. These results demonstrate the advantages of the Laplacian electrode module.

## 1. Introduction

Recent advances in information and communication technologies such as the Internet and e-mail have led to their world-wide use. As a consequence, the so-called “digital divide” between persons able to easily use these technologies and others who cannot, especially those with physical disabilities, has increased [1]. There are, therefore, urgent needs for developing interfaces that can help individuals with physical disabilities to operate pointing and/or character-input devices (see e.g., [2,3]). Because conventional human-computer interfaces work basically in response to physical motions (a good example for this is handwriting as input, see e.g., [4]), biosignals which eventually yield the motions can also be used for such interfaces [5]. In particular, electromyogram (EMG) signals have often been studied and actually used as control signals for artificial limb prostheses [6–9], robot hands [10], a manipulator [11] and a pointing device [12]. This may be because the EMG signal contains useful information about motion intent, muscle movement, muscle force, and muscle impedance.

The use of conventional interfaces employing wired electrodes and a wired amplifier is limited by the length of the connecting wires, and is restricted to persons who never suffer from involuntary limb actions. Therefore persons with physical disabilities such as cerebral palsy have little or no access to biosignal-based human-computer or human-machine interfaces. In addition, deaf-blind people cannot use a computer and accordingly, also have only little access to human interfaces. One method to communicate with those people is finger Braille, which uses tactile sensation. Some deaf-blind people can communicate with others through a finger Braille interpreter. Because finger Braille uses a code similar to Braille, it is relatively easy to develop an electro-mechanical device for finger Braille. Actually, there exist some studies aiming at developing a system which can automatically convert a text into tactile information and *vice versa* and function as interpreter [13,14]. To communicate more smoothly using such a system, understanding the prosody (rhythm and stress) of natural finger Braille is important.

In this paper, we have proposed a compact wireless Laplacian electrode module for EMG and apply it to character-input interface and evaluation of finger Braille typing. In our previous study, we proposed the Laplacian electrode configuration for EMG recording [15]. However, the developed system in [15] was not wireless, and it was not validated through any actual application. Although we confirmed synchronous firings with the Laplacian and conventional EMGs, we did not address the difference in characteristics as input for human interface. Our primary aim here was to demonstrate that the Laplacian EMG was better than the conventional EMG in actual human interface applications.

The Laplacian electrode configuration was easily implemented in the wireless module because the measuring electrodes can be accommodated together in a small area. In addition, as mentioned later, EMGs obtained with the Laplacian configuration were expected to be sensitive to the firing of the muscle directly beneath the measurement site. A character-input interface was developed using the electrode module with combination of an EMG-mouse click converter circuit and scanning cursor software. Investigations on the performance of the character-input interface were carried out in healthy male subjects. One of the purposes of this investigation was to verify the usability of the electrode module. In the application to finger Braille typing, four electrode modules were attached to an interpreter, two on each forearm, to detect finger movements during typing. One finger Braille interpreter participated in an experiment for finger movement detection. In this experiment, we also examined whether the four modules were able to work properly in parallel.

## 2. Theoretical Basis of Body Surface Laplacians

Use of the body surface Laplacian was first proposed by Hjorth for electroencephalogram (EEG) recording in 1975 [16], and was later applied to EMG derivation by Reucher *et al.* in 1987 [17,18], and to electrocardiogram (ECG) measurement by He and Cohen in 1992 [19]. While the use of the surface Laplacian has been growing for current source estimation in EEG and ECG measurements, it has not been widely used for EMG measurement, especially in relation to human-computer or human-machine interfaces.

Considering a local orthogonal coordinate system (*x, y, z*) with an origin at a point on the body surface where the *z* axis is orthogonal to the body surface, the Laplacian EMG, *L*_S_, is defined by applying a Laplacian operator to the body surface potential *ϕ*, as follows:

(1) $$\begin{array}{l} L_{s}=-{\nabla_{xy}}^{2}\phi \\ =-\nabla_{xy}\cdot \nabla_{xy}\phi \equiv -\left(\frac{\partial^{2}\phi }{\partial x^{2}}+\frac{\partial^{2}\phi }{\partial y^{2}}\right). \end{array}$$

If the body is assumed to be a linear, isotropic and piecewise homogeneous conductor, the gradient of the electrode potential *ϕ* is proportional to the electrical field *E*:

(2) −∇ϕ=E.

On the other hand, Ohm's law requires:

(3) $$E=\frac{1}{\sigma }J$$

where *J* is the current density and *σ* is the electrical conductivity. Combining Equations (1-3) gives

(4) $$\begin{array}{l} L_{s}=\nabla_{xy}\cdot E_{xy}\\ =\nabla_{xy}\cdot \frac{1}{\sigma }J_{xy}=\frac{1}{\sigma }\left(\frac{\partial J_{x}}{\partial x}+\frac{\partial J_{y}}{\partial y}\right). \end{array}$$

The following equation shows that the divergence of the total current vanishes under quasi-static conditions:

(5) $$\nabla \cdot J=\frac{\partial J_{x}}{\partial x}+\frac{\partial J_{y}}{\partial y}+\frac{\partial J_{z}}{\partial z}=0$$

where ∇ · is the divergence operator. By putting Equation (5) into Equation (4)*L*_s_ can be expressed as:

(6) $$L_{s}=\frac{1}{\sigma }\left(-\frac{\partial J_{z}}{\partial z}\right)=-\frac{1}{\sigma }\left(\frac{\partial J_{z}}{\partial z}\right).$$

Thus the Laplacian EMG signal is negatively proportional to the normal derivative of the normal component of the current density at the body surface [19]. Therefore, the Laplacian EMG is considered to be sensitive to the firing of the muscle directly beneath the measurement site. Accordingly, the signal is potentially useful as an input for human interfaces, because it is less susceptible to interference caused by the activity of neighboring muscles than the conventional EMG signal.

**Figure 1.**
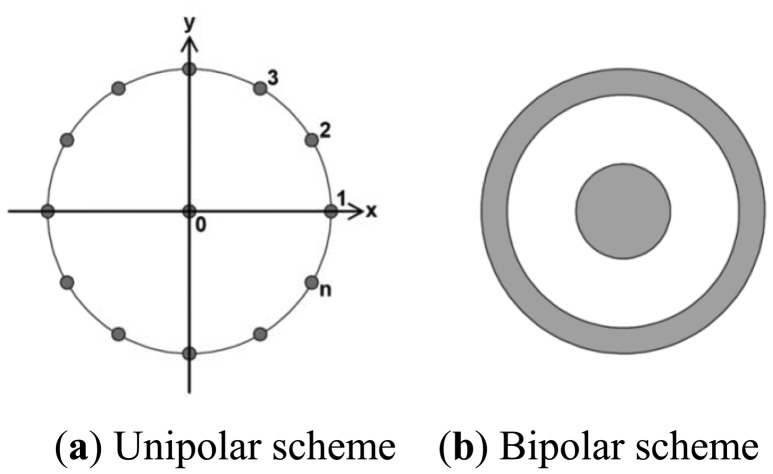
Schematic diagrams of measuring electrodes for deriving a surface Laplacian potential. (**a**) The unipolar electrode configuration. The potential data are collected at *n* points over small circles surrounding the observation point 0. (**b**) The bipolar concentric electrode configuration composed of a conductive disk at the center and a conductive ring surrounding the central disk.

There are several electrode configurations that can be used to yield the Laplacian derivation. The most widely used configurations are unipolar and bipolar schemes (Figure 1) [20,21]. In the unipolar scheme, the Laplacian potential *L*_0_ at observation point 0 can be estimated as:

(7) $$L_{0}≅\frac{4}{r^{2}}\left(\phi_{0}-\frac{1}{n}\sum_{i=1}^{n}\phi_{i}\right)$$

where *ϕ*_i_ represents the potential at one of the surrounding points, *r* is the radius of the circle, and *n* is the number of points surrounding the circle. In the bipolar scheme, the Laplacian potential can be expressed by:

(8) $$L_{0}≅\frac{4}{b^{2}}\left(\phi_{0}-\frac{1}{2\pi b}\oint \phi dl\right)$$

where the integral is taken around a circle of radius *b*[19].

**Figure 2.**
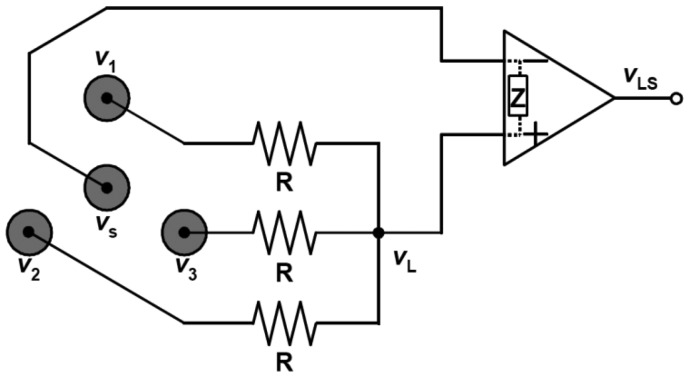
MacKay's configuration of electrodes and a circuit connected to them for deriving an approximate surface Laplacian potential.

We can implement the electrodes together in a small area with both configurations. This feature is preferable for the implementation of electrodes in a single module, and thus for fabrication of an active wireless device that functions not only as an electrode module but also as a device for amplifying, filtering and transmitting the detected signal. In the developed wireless Laplacian electrode module, we employed a configuration proposed by MacKay (Figure 2) [22], where three positive electrodes were aligned on each apex of an equilateral triangle and one negative (source) electrode was placed at the center of the triangle. If the input impedance, *Z*, of the operational amplifier is far larger than the resistance, *R*, in Figure 2, then the current through *Z* is negligible. Then, we can express the total amount of current passing through the resistance according to Kirchhoff's law as follows:

(9) $$\frac{v_{1}-v_{L}}{R}+\frac{v_{2}-v_{L}}{R}+\frac{v_{3}-v_{L}}{R}=\frac{v_{L}-v_{s}}{Z}≅0.$$

Therefore:

(10) $$v_{L}≅\frac{v_{1}+v_{2}+v_{3}}{3}.$$

Then *v*_LS_ can be described using the gain *A*_v_ of the amplifier by:

(11) $$\begin{array}{l} v_{LS}≅A_{v}\left(v_{s}-\frac{v_{1}+v_{2}+v_{3}}{3}\right)\\ =A_{v}\left(v_{s}-\frac{1}{3}\sum_{i=1}^{3}v_{i}\right). \end{array}$$

Thus, the adopted configuration is a special case of the unipolar scheme when *n* equals 3 in Equation (7).

## 3. Materials and Methods

### 3.1. Wireless Electrode Module

**Figure 3.**
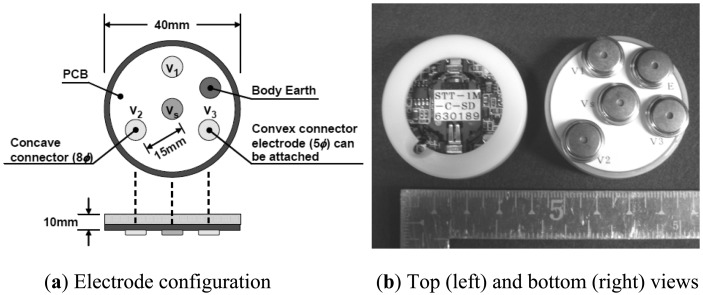
Configuration and appearance of the developed wireless electrode module. (**a**) Configuration and (**b**) Top and bottom views.

We developed a compact wireless electrode module for the Laplacian EMG measurement. In the module, we implemented the electrodes, an amplifier, a filter and a transmitter. The electrodes were aligned according to MacKay's configuration [22]. Figure 3 shows the configuration and appearance of the developed wireless module. The specifications of the module are listed in Table 1. As can be seen in Figure 3 and Table 1 the device is nearly equal in size to a wristwatch and light enough to be worn. The input impedance of the module was 100 MΩ to obviate skin preparation and the use of conductive gel or paste between the skin and the electrodes. The gain of the amplifier was designed to be in a range between 2,000 and 4,000. This gain is larger than that of a commercially available amplifier for EMG, whose gain is usually 1,000. The larger gain was selected because the amplitude of signals detected with the Laplacian configuration is smaller than that with the conventional configuration. The cut-off frequency of the high-pass filter was set to 5 Hz to reduce the influence of motion artifacts. In addition to a high pass filter, the commercially available EMG amplifiers also employ low pass filters (LPFs) to remove unnecessary higher frequency components, and most of the EMG amplifiers have two or more LPFs so that users can choose an appropriate cut-off frequency for their environment. The developed device employs two LPFs, whose cut-off frequencies are 500 Hz and 1 kHz.

A commercially available digital frequency modulation transmitter (TMB-012, Japan Radio Co., Tokyo, Japan), which uses a frequency-shift keying (FSK) scheme, is employed for the wireless communication. Although Bluetooth has been becoming more and more common for medical applications, we choose the transmitter based on its safety performance in medical facilities and economic efficiency. The 300 MHz band, which is allowed to use without a license under the regulation on extremely low power radio station in Japan, is employed because of the following reasons: (1) it has the widest band width of 1 MHz and enables (2) multiple channel communication using FSK and (3) relatively long range communication.

### 3.2. Character-Input Interface

#### 3.2.1. EMG-Mouse Click Converter Circuit

**Figure 4.**
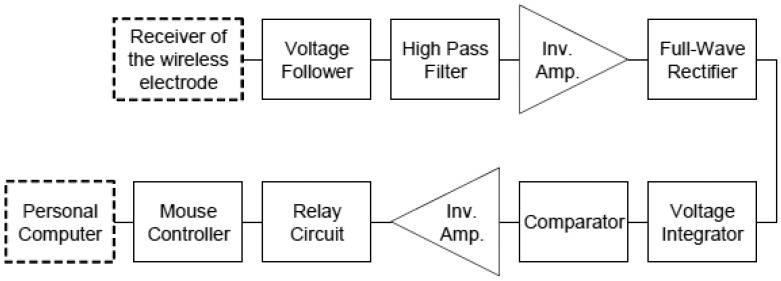
Block diagram of the assembled EMG-click converter circuit.

In order to convert the EMG signal into a click signal of a computer mouse, we assembled an EMG-mouse click converter circuit. Figure 4 shows its block diagram. First, the wireless transmitted EMG signal was fed to a voltage follower to prevent voltage loss and then high-pass filtered with a cut-off frequency of 5 Hz to reduce the baseline fluctuation. Next the filtered signal was rectified by a full-wave rectifier and converted to an integrated EMG (IEMG) signal. Finally the IEMG was compared with a threshold of a comparator and then transformed into a pulse signal when the IEMG exceeded the threshold. Threshold hysteresis was employed to prevent chattering. The threshold could be altered by a variable resistor in the comparator. Once the pulse signal was input to a relay circuit (G6C-211P-US, Omron, Kyoto, Japan), the two terminals of a mouse controller, which was connected to a personal computer via a universal serial bus (USB), were short-circuited and a click signal was generated by the controller. The controller was taken out from a commercially available mouse.

#### 3.2.2. Character Input Software with Scanning Cursor

**Figure 5.**
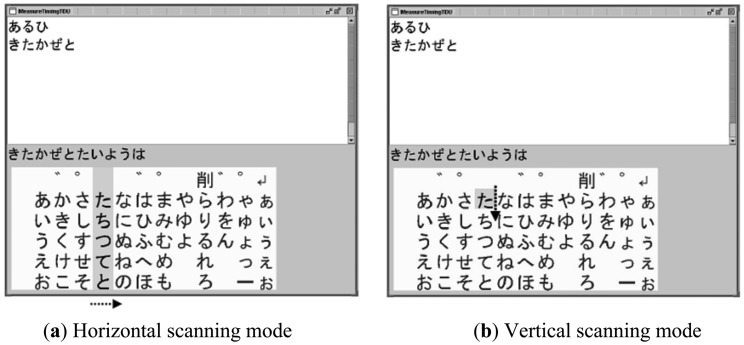
Execution screens of the character-input software with a scanning cursor. (**a**) Horizontal and (**b**) Vertical scanning modes. For those who are not familiar with Japanese characters, the main idea presented in this figure was reproduced in alphabets in Figure A1.

We modified a software program developed in [23,24] so that it assists disabled persons to input characters by contracting one of their muscles. Figure 5 shows examples of screens during the software execution. The user interface of the software was based on a standard matrix of Japanese “kana” characters and an active cursor. The standard matrix is familiar to the Japanese and used in many commercial communication aids in Japan. The active cursor scanned the characters horizontally or vertically depending on its scanning mode. In the horizontal scanning mode, a column cursor masked with a gray color moved from the left to the right recursively in the matrix as shown in Figure 5(a). The column stopped when a mouse click signal was generated as a result of a muscle contraction and then the scanning mode was changed to the vertical one. In this mode, a square row cursor with a mask indicating one character moved from the top to the bottom in the previously selected column recursively (Figure 5(b)). For those who are not familiar with Japanese characters, the main idea presented in this figure was reproduced in alphabets in Supplemental Figure 1. A character in the row cursor was entered and displayed in the window of the software when another mouse click signal was generated. After the second click, the scanning mode returned to the horizontal one. In this way, the user could enter one character by two mouse click signals, *i.e.* two contractions of a muscle in response to the movement of the cursor.

#### 3.2.3. Character Input Experiments

We investigated whether characters could be input as desired using the developed interface system. The wireless electrode module was attached to various sites on the body surface: (a) the masseter, (b) trapezius, (c) *anterior tibialis* and (d) *flexor carpi ulnaris* muscles. One male volunteer aged 24 participated in the experiments. No skin preparation was performed and no adhesive paste or gel was used for the attachment. The subject was instructed to input 115 Japanese characters by (a) clenching the teeth, (b) raising the head, (c) lowering the tip of the toe, or (d) flexing the middle finger. In the experiments, the entered characters were logged in the personal computer, in which the character input software was installed. Also, the Laplacian EMG and pulses generated by the EMG-mouse click converter were recorded with a data acquisition system (MP-150, Biopac Systems, Goleta, CA, USA). We measured the time required to input the 115 characters including corrections of characters. An error rate was calculated as the number of corrections divided by 115.

As a benefit of the Laplacian electrode configuration, we expected it to reduce a risk of secondary disabling due to overwork of the muscle from which the EMG signal was derived for the interface. In order to evaluate the muscle activity required for character input, we compared the IEMGs with the Laplacian and conventional configurations. Eight male subjects without physical disabilities, aged between 21 and 29, participated in the experiment. The wireless module was attached to the *flexor carpi ulnaris* muscle of the right forearm. For the conventional EMG recording, two electrodes with lead wires were attached on both sides of the wireless module along the muscle, and a reference electrode was placed at the distal end of the radius of the same forearm. The Laplacian and conventional EMG signals were used as the input signal in Conditions 1 and 2, respectively. In both conditions, the subjects were instructed to input a sentence consisting of 115 characters using the system by flexing the forefinger or the middle finger in one trial. Five trials were conducted under each condition and the sequence of the conditions was randomized. The subjects were allowed sufficient practice before each trial. To evaluate the muscle activity during the use of the interface, the IEMG was calculated from the conventional EMG in both conditions so that the conditions except the electrode configuration were identical. The time constant was 0.016 s. The mean IEMGs in both conditions were compared statistically.

### 3.3. Detection of Finger Movement during Finger Braille

Understanding the prosody (rhythm and stress) of natural finger Braille is important to aid smooth communication using a computer-based finger Braille system. We, therefore, investigated whether prosodic information can be obtained from EMG recorded with the electrode module. In this experiment, we also aimed to examine whether multiple electrode modules were able to work properly in parallel.

One finger Braille interpreter, who has worked as an interpreter for more than 20 years, participated in an experiment for finger movement detection. He gave written informed consent. The interpreter translated three news articles of about 130 characters in Japanese (when read it took about 20 s) into finger Braille. The same translation was performed three times, and the average translation rate was 6.37 characters/s.

**Figure 6.**
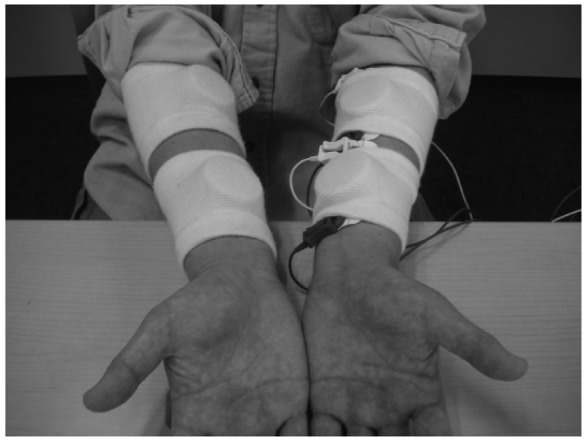
Arrangement of the wireless electrodes and the disposable electrodes for detection of finger movements during finger Braille typing. Four Laplacian modules, two on each flexor carpi ulnaris muscle, were attached to the finger Braille interpreter.

As shown in Figure 6, four electrode modules were attached to the interpreter, two on each *flexor carpi ulnaris* muscle, to detect finger movements during finger Braille typing. No skin preparation was carried out for the electrode module. For a comparison, two conventional disposable electrodes (F-150S, Nihon Kohden, Tokyo, Japan) were also placed on the left *flexor carpi ulnaris* muscle. For the conventional electrodes, the earth electrode was placed on the left elbow, and a commercially available amplifier (EMG100C, Biopac Systems, Goleta, CA, USA) was used under the following conditions: the gain was 1,000 and the pass band was between 1 Hz and 5 kHz. Then the filtered EMG signal was rectified by a full-wave rectifier and converted to an IEMG signal. The time constant was 0.016 s. Based on the IEMG, the timing of finger Braille typing was determined with a threshold level: 10% and 20% of the maximum IEMG level. An error rate in detecting the typed Braille number was calculated to evaluate the performance. One of the purposes of this experiment was to demonstrate that multiple wireless modules could be used simultaneously.

## 4. Results

### 4.1. Character Input Experiments

**Figure 7.**
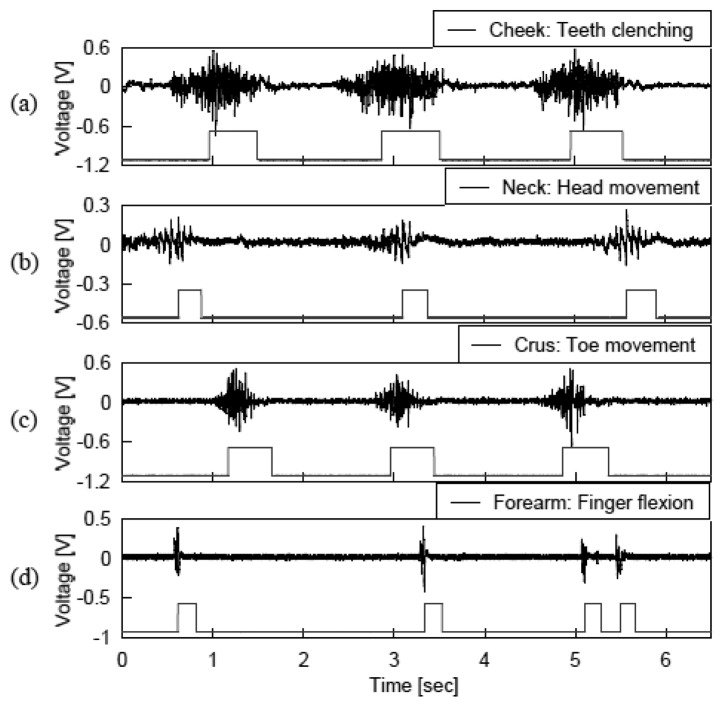
Recordings of the Laplacian EMG signals obtained from various sites with muscle contractions and corresponding pulse signals generated by the EMG-click converter circuit. (**a**) Masseter, (**b**) trapezius, (**c**) *anterior tibialis*, and (**d**) *flexor carpi ulnaris* muscles.

Figure 7 shows recordings of the Laplacian EMGs and the corresponding pulse signals generated by (a) clenching the teeth, (b) raising the head, (c) lowering the tip of the toe, or (d) flexing the middle finger. The figure indicates that the muscle contraction was stably converted to the mouse click signal, and thus the sentences were smoothly entered using the developed system. The average time required to input the 115 characters was about 600 s. This corresponds to a rate of 0.19 characters/s. On average, the subject made one or two corrections in each trial and thus, the error rate was 1.7%. The system can be used by attaching the wireless module on the target muscle without skin preparation. Accordingly, the preparation time and efforts required for setting the electrode module were less than those for the conventional electrodes.

**Figure 8.**
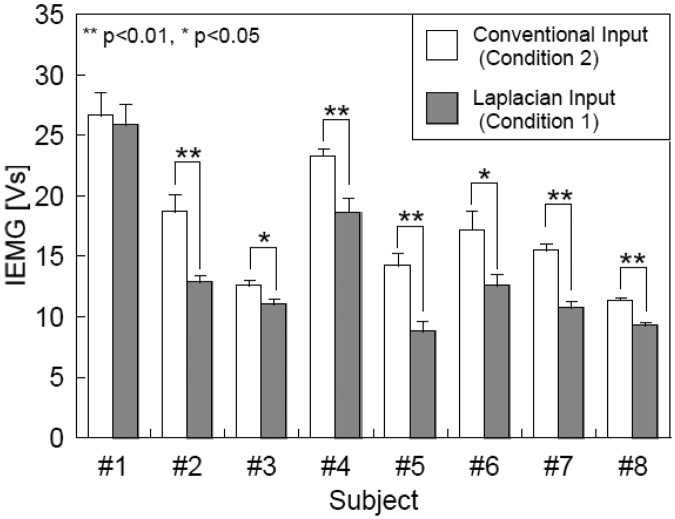
Comparison between the mean IEMGs obtained with the conventional and Laplacian electrodes.

As shown in Figure 8, the mean IEMGs were decreased in all subjects when the Laplacian EMG was used as the input signal (Condition 1) than those with the conventional EMG (Condition 2). The decreases were significant (p < 0.05) in seven out of the eight subjects. Because the IEMGs in both conditions were calculated from the conventional EMGs measured at the same site with the same electrodes, the decreases in IEMG were attributable to the changes in finger motion during the operation of the interface.

### 4.2. Finger Movement Detection during Finger Braille Typing

**Figure 9.**
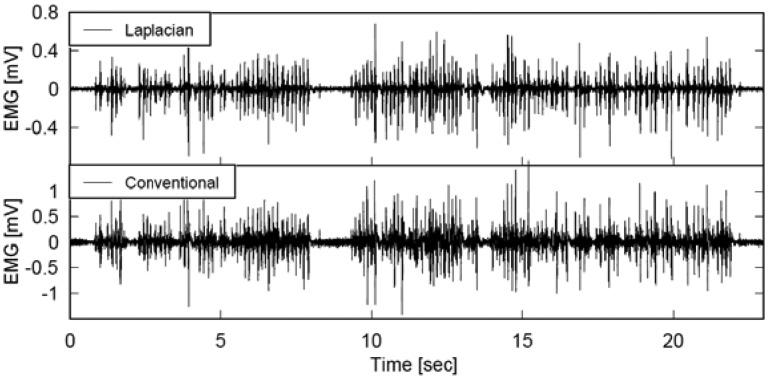
Typical recordings of the Laplacian (**top**) and conventional (**bottom**) EMGs during real time interpretation of broadcast news to finger Braille.

**Figure 10.**
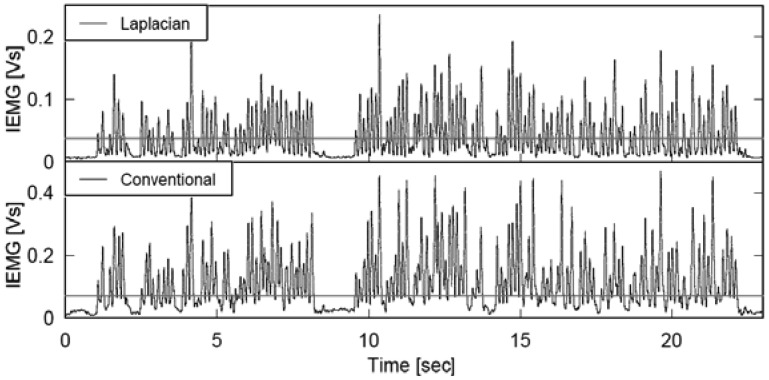
IEMGs with the Laplacian (**top**) and conventional (**bottom**) EMGs during real time interpretation of broadcast news to finger Braille.

Figures 9 and 10 illustrate the EMG and IEMG during real time interpretation of broadcast news to finger Braille. Although the graphs in each figure seem to be similar at a glance, a thorough comparison of the two graphs in each figure revealed that the signal amplitude near the 0 mV level was smaller with the Laplacian electrode module, and the smaller power enabled us to detect the timing of finger movement more clearly. The error rate in detecting the typed Braille number was calculated to quantitatively investigate this. When the threshold was set at 10% of the maximum IEMG level, the error rates with the Laplacian electrode module were 4.6% and 6.8% at the distal and proximal sides, respectively. On the other hand, they were 28.8% and 25.9% for the conventional electrode. With a threshold of 20%, the rates were 3.0% and 5.4% for the developed electrode module while they were 8.1% and 6.2% for the conventional electrode.

## 5. Discussion

### 5.1. Wireless Laplacian Electrode Module

The performance of the developed wireless Laplacian electrode module was investigated in two human interface applications. The experiments of character input interface and finger Braille typing validated the developed module is applicable to human interface applications. One of the advantages of the module is that it will not interfere with any movement of the user because it employs wireless communication. Another advantage is that EMG obtained with it is expected to be sensitive to the firing of the muscle directly beneath the measurement site. In addition, the high input impedance of the amplifier in the module obviates the need for conventional skin preparation and the use of conductive gel or paste and thus discomfort caused by these. Accordingly, attachment of the wireless module takes only about 30 s, and the skin in the attached area is less irritated and/or inflamed. As demonstrated in the finger Braille experiment, the four modules functioned properly in parallel. This indicates that multiple modules can be used simultaneously. These features are desirable for technical aids as well as human-computer interfaces that are likely to be used for long hours every day.

### 5.2. Character Input Experiments

In addition to the four muscles reported above, we repeated the character input experiments with the wireless module placed at the thigh or *brachium* (data not shown). Similar to the result in Figure 7, the muscle contraction was stably converted to the mouse click signal. These results strongly suggest that the developed system can be applicable to wide variety of muscles. This broad applicability of the developed system is advantageous for technical aids for persons with physical disabilities because affected parts of the body vary considerably one person to another.

Figure 8 indicates the mean IEMGs were decreased significantly in the seven subjects when the Laplacian EMG was used. These results imply that the required muscle activity for character input was smaller with the Laplacian EMG compared to the conventional EMG. The Laplacian electrode configuration can record an EMG signal from just beneath the measuring site [19] and accordingly, the Laplacian EMG is less sensible to activities of muscles around the one to be investigated. It was, therefore, highly likely that the operators were able to input characters with smaller muscle contractions. Approximately 80% of computer users, whose jobs involve repetitive wrist movements and awkward hand positions, exhibit musculoskeletal dysfunction [25,26]. Accordingly, users of EMG-based computer interfaces may also develop similar dysfunction due to repetitive contractions of a specific muscle for character input. Because the developed interface requires smaller muscle contractions, it is desirable to reduce a risk of secondary disabling due to overwork of the muscle from which the EMG signal was derived. This is another advantage of the Laplacian electrode module.

### 5.3. Finger Movement Detection during Finger Braille Typing

The finger Braille experiment demonstrated the proper functioning of multiple modules used simultaneously. A single receiver was able to receive signals from two transmitters simultaneously by using slightly different frequency. Accordingly, two receivers were used in this experiment. The experimental results indicate that the receivers were able to process the wireless transmitted EMG without any crosstalk even when used in parallel.

One advantage of measuring prosodic information using EMG is that the method can be used during actual communication by finger Braille. A pressure sensor can be used to detect the finger movement [27]. However, the pressure sensor can hardly measure the movement during the actual finger Braille communication because the pressure sensor is usually made of a hard material and thus, the finger movement of a person cannot be sensed by the other. Although the interpreter did not communicate with a deaf-blind in the experiment in this study, the EMG method will not interfere, in theory, with communication by finger Braille.

## 6. Conclusions

In this study, we have developed a compact wireless Laplacian electrode module for EMG. The performance of the developed electrode module was investigated in two human interface applications. First, the module was used as a part of a character-input interface system, which consisted of the module, an EMG-mouse click converter circuit and software. Then the module was used for detection of finger movements during finger Braille typing. The evaluation of the character-input interface indicated that characters could be input stably by contraction of (a) the masseter, (b) trapezius, (c) *anterior tibialis* and (d) *flexor carpi ulnaris* muscles. This wide applicability is desirable as a technical aid for persons with physical disabilities because the disability differs one to another. Experiments for finger movement detection showed that each finger movement was more clearly detectable when comparing to EMG recorded with conventional electrodes, suggesting that the Laplacian electrode module is more suitable for detecting the timing of finger movement during typing. These results demonstrated advantages of the Laplacian electrode module.

## Figures and Tables

**Table 1.** Specifications of the developed wireless electrode module.

**Item**	**WirelessModule (Transmitter)**	**Receiver**
Gain	2,000–4,000 (66–72 dB)	-
High-pass filter	5 Hz	-
Low-pass filter	-	500 Hz (on)/1 kHz (off)
Weight	11 g	140 g
Dimension	40 mm (ϕ) × 10 mm	80 mm × 125 mm × 30 mm
Power supply	Coin-shaped lithium ion battery (CR2032), 3 V	AC adapter or battery (006P), DC 9 V
Current	2 mA	<20 mA

**Figure A1.**
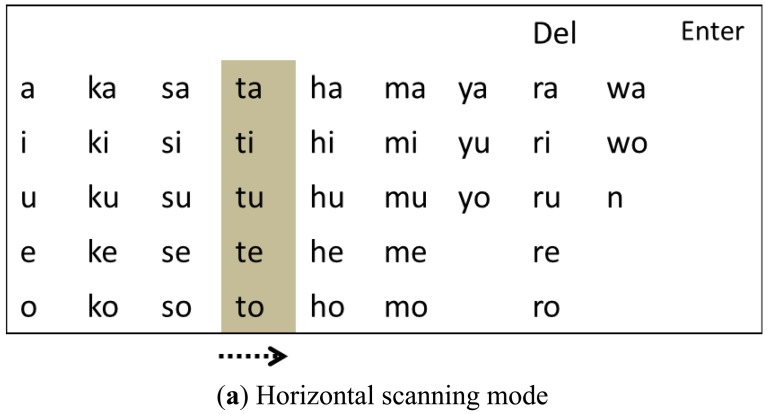

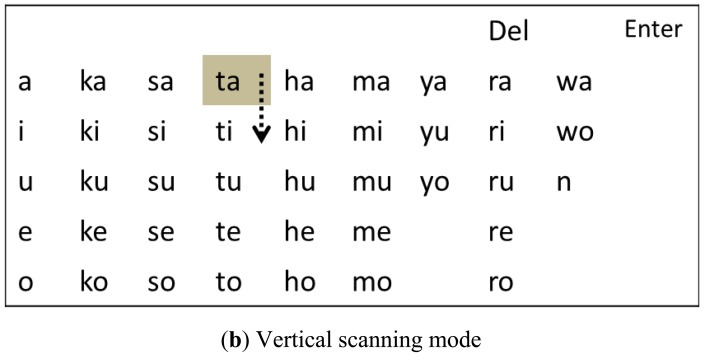
Two scanning modes of the character-input software with a scanning cursor. (**a**) Horizontal and (**b**) Vertical scanning modes. The information presented in this figure was reproduced from Figure 5.
